# The cingulate–mitochondrial–resonome axis: a conceptual framework integrating rehabilitation, interoception, and affective–motor processing in canine chronic pain

**DOI:** 10.3389/fpain.2026.1807872

**Published:** 2026-10-09

**Authors:** Holly Pollard-Wright, Alexandra Klomhaus

**Affiliations:** 1 Veterinary Kinetics Rehab Inc, Torrance, CA, United States; 2 Division of General Internal Medicine and Health Services Research, UCLA David Geffen School of Medicine, Los Angeles, CA, United States

**Keywords:** autonomic regulation, bioenergetics, canine rehabilitation, cingulate cortex, consciousness, electromagnetic fields, fascia, FoK–FIP theory

## Abstract

Chronic musculoskeletal disease in *Canis lupus familiaris* (dogs) is associated not only with biomechanical dysfunction but also with alterations in autonomic regulation, affective processing, movement behavior, and chronic pain adaptation. Current rehabilitation models primarily emphasize peripheral tissue healing and biomechanics while placing less emphasis on interactions among interoception, nociception, autonomic regulation, and affective-motor processing. The objective of this Hypothesis and Theory article is to present the Cingulate–Mitochondrial–Resonome Axis (CMRA), a systems-level framework proposing that rehabilitation acts in part by restoring the coherence and organization of interoceptive signaling (the resonome), thereby influencing cingulate-mediated affective, autonomic, nociceptive, and motor regulation. Building conceptually upon the Feelings of Knowing–Fundamental Interoceptive Patterns (FoK–FIP) theory, the CMRA framework proposes that distributed interactions among connective tissue, autonomic, sensorimotor, and bioenergetic systems contribute to organism-level adaptation during rehabilitation. The central hypothesis is that restoration of interoceptive coherence serves as a candidate mediating variable linking peripheral rehabilitation interventions to central neurocognitive and behavioral change. Within this framework, functional improvements may occur despite persistent structural pathology, and rehabilitation-induced effects may extend beyond predictions made by strictly local biomechanical models. These predictions distinguish the CMRA framework from conventional structure-centered rehabilitation models and generate experimentally testable hypotheses for future investigation. The manuscript further proposes that rehabilitation-associated physiological changes may be reflected by multimodal measures including infrared thermography, quantitative gait analysis, and validated behavioral assessments. While these measures are not specific to CMRA and may also reflect established mechanisms such as altered biomechanics, reduced inflammation, central sensitization, and predictive processing, the framework predicts coordinated changes across multiple domains consistent with improved interoceptive organization. The CMRA framework is presented as a testable theoretical model rather than an established mechanistic explanation. Although the proposed role of large-scale endogenous electromagnetic organization remains speculative and incompletely validated, the framework generates falsifiable predictions regarding rehabilitation-associated changes in behavior, autonomic regulation, gait function, and chronic pain adaptation across species.

## 1 Introduction

Chronic musculoskeletal dysfunction—including osteoarthritis, tendinopathy, myofascial pain syndromes, and spinal instability—produces effects that extend beyond structural tissue pathology alone. In both humans and companion animals, chronic pain states are associated with measurable alterations in motor behavior, autonomic regulation, affective processing, motivational drive, and social engagement (1–5). Within canine rehabilitation, owners frequently report behavioral changes including decreased willingness to perform previously routine movements, reduced playfulness, altered environmental engagement, increased irritability, and withdrawal from social interaction (3). These observations may reflect overlapping contributions from nociceptive input, pain-anticipatory learning, central sensitization, protective motor behavior, and affective-motivational processes involving supraspinal pain networks (6–9).

The epidemiological burden of these disorders is incompletely characterized in dogs and varies with diagnostic criteria and study population. In a large UK primary-care population, the annual period prevalence of diagnosed appendicular osteoarthritis was estimated at 2.5% (10). Myofascial trigger points have also been reported frequently in dogs with osteoarthritis, although available canine studies have involved selected clinical populations rather than population-based surveillance (11). Available epidemiological estimates for canine tendinopathy, myofascial pain syndromes, and spinal instability are derived largely from specific clinical, anatomical, or breed-defined populations, limiting direct comparison of population-level burden across these broad diagnostic categories.

The objective of this Hypothesis & Theory article is to introduce the Cingulate–Mitochondrial–Resonome Axis (CMRA), a systems-level conceptual framework proposing that rehabilitation may act in part by restoring the coherence and organization of interoceptive signaling (the resonome), thereby influencing cingulate-mediated affective, autonomic, nociceptive, and motor regulation relevant to chronic pain adaptation and functional recovery.

Although substantial evidence characterizes interoception, autonomic regulation, mitochondrial function, connective tissue biology, and central nervous system processing as physiologically interconnected domains (12–16), these mechanisms are typically investigated in relative isolation. Consequently, current rehabilitation theory lacks a unified systems-level framework explaining how physiotherapy may simultaneously influence these interconnected processes during recovery from chronic musculoskeletal disease.

The proposed framework addresses this gap by positioning the cingulate cortex as an integrative hub linking interoceptive processing, autonomic regulation, mitochondrial bioenergetics, fascial mechanotransduction, and motor adaptation within a unified systems model. Central to the framework is the hypothesis that rehabilitation-associated changes in the coherence and organization of interoceptive signaling (the resonome) serve as a candidate mediating variable linking peripheral physiological adaptation to central neurobehavioral change. Rather than reviewing the literature comprehensively, this article synthesizes representative evidence into a unified conceptual framework that generates experimentally testable predictions and falsification criteria for future research and rehabilitation practice.

Current veterinary rehabilitation paradigms appropriately emphasize peripheral tissue healing, inflammatory modulation, biomechanical correction, neuromuscular reconditioning, and restoration of functional mobility (10, 11, 17, 18). These approaches often improve joint mobility, muscle strength, gait performance, and overall functional capacity. However, they less directly address how chronic pain states influence affective regulation, autonomic responsiveness, motivational behavior, and higher-order motor decision-making. Consequently, the mechanistic relationship between peripheral musculoskeletal rehabilitation and central neurobehavioral adaptation remains incompletely characterized within veterinary rehabilitation medicine.

Emerging evidence from both human and non-human animal research suggests that chronic musculoskeletal pain involves distributed interactions among nociceptive processing, autonomic regulation, interoception, affective state, and motor planning networks rather than isolated peripheral tissue pathology alone (1–9). Brain regions including the cingulate cortex appear to play important roles in integrating these processes and shaping behavioral responses to pain, uncertainty, effort, and anticipated movement-related outcomes (7, 8, 18). These observations support the need for more neurobiologically integrated rehabilitation frameworks capable of incorporating interactions among peripheral tissue state, central pain processing, autonomic regulation, and affective-motor behavior. Collectively, these observations suggest that integrating these interacting systems into a single conceptual framework may improve mechanistic understanding, guide hypothesis-driven research, and inform future rehabilitation strategies in veterinary medicine. Because many of the neurophysiological mechanisms discussed are evolutionarily conserved across mammalian species, findings from human and experimental animal studies are incorporated where appropriate to inform the proposed framework while emphasizing its intended application to canine rehabilitation.

The present manuscript introduces the Cingulate–Mitochondrial–Resonome Axis (CMRA) as a testable systems-level hypothesis proposing that rehabilitation may act in part through restoration of interoceptive coherence, thereby influencing cingulate-mediated affective, autonomic, nociceptive, and motor regulation. The sections that follow develop the biological rationale for this framework, examine its relationship to existing theories, and outline experimentally testable predictions and falsification criteria that may guide future rehabilitation research.

### 1.1 Chronic pain, sensitization, and pain vs. nociception

Chronic pain is increasingly recognized as a multidimensional neurobiological state involving sensory, affective, cognitive, autonomic, and behavioral components rather than a simple reflection of ongoing peripheral tissue injury (19–22). In both humans and companion animals, persistent musculoskeletal dysfunction may contribute to long-term alterations in central nervous system processing involving attention, interoceptive representation, motivational-affective processing, and context-dependent behavioral responses to anticipated movement or discomfort (12, 13, 23–25).

Importantly, nociception and pain are not synonymous phenomena. Nociception refers to the neural encoding and transmission of potentially tissue-damaging stimuli, whereas pain represents a subjective and integrative experience shaped by sensory input, affective state, prior learning, contextual interpretation, autonomic state, and cognitive appraisal (26–29). As a result, behavioral responses to chronic pain may persist even when peripheral tissue pathology alone does not fully account for observed functional impairment or affective changes (26–29). Chronic pain states are also associated with alterations in emotional processing, attentional allocation, affective pain processing, and cognitive control (30–33). These changes may contribute to altered behavioral responses relevant to rehabilitation outcomes. Consequently, rehabilitation outcomes may reflect not only improvements in peripheral tissue function but also adaptive changes within distributed central pain and affective-processing networks.

This broader neurobiological perspective provides the biological rationale for the Cingulate–Mitochondrial–Resonome Axis (CMRA). Building upon these established concepts, the following sections present the CMRA as a systems-level conceptual framework integrating interoception, autonomic regulation, mitochondrial bioenergetics, affective processing, and motor behavior to generate experimentally testable hypotheses relevant to rehabilitation.

### 1.2 The cingulate Cortex as an integrative hub for affect, nociception, and motor control

The cingulate cortex—particularly its anterior (ACC) and midcingulate (MCC) subdivisions—is increasingly recognized as an important integrative network involved in affective processing, nociceptive evaluation, autonomic regulation, interoceptive representation, and context-dependent motor behavior (22, 34–37). In both humans and non-human animals, the cingulate cortex receives convergent input from nociceptive pathways, proprioceptive systems, viscerosensory networks, and limbic structures including the amygdala, hippocampus, and ventral striatum (22, 38–42). These distributed connections may enable dynamic integration of internal physiological state, anticipated effort, emotional salience, and behavioral prioritization during movement, recovery, and adaptation to chronic pain states.

The ACC has been strongly associated with affective dimensions of pain processing, motivational salience attribution, conflict monitoring, autonomic arousal, and anticipation of aversive or effort-related outcomes (7, 22–24, 28–33, 38, 39). Importantly, pain perception is not solely determined by nociceptive input intensity, but also by affective interpretation, prior experience, autonomic state, and contextual prediction. Functional neuroimaging and lesion studies in humans demonstrate that ACC activity correlates more consistently with the unpleasantness and emotional salience of pain than with sensory intensity alone (7, 8, 22, 26, 27, 32–36). The ACC also participates in pain modulation through interactions with descending pain-control systems (7, 27, 33).

The MCC, in contrast, appears to play a greater role in action selection, movement initiation, gait-related behavioral adaptation, effort allocation, and motor planning under conditions of uncertainty or anticipated discomfort (22, 37–41). Together, ACC and MCC networks may contribute to behavioral adjustments that occur during chronic pain states, including altered movement patterns, protective motor strategies, reduced exploratory behavior, and changes in motivational engagement.

The amygdala also contributes importantly to affective processing, threat-related responses, and pain-related behavior (7, 8, 22, 27, 33). Interactions between the amygdala and cingulate cortex may influence behavioral responses to anticipated pain, environmental stressors, frustration, and movement-related avoidance behaviors. In both human and non-human animal models, chronic pain states have been associated with altered functional interactions among cingulate, amygdalar, autonomic, and sensorimotor networks (7, 12, 13, 22, 27, 33, 43).

In canine rehabilitation contexts, chronic musculoskeletal dysfunction may therefore contribute not only to peripheral biomechanical impairment but also to broader neurobehavioral and affective-motivational changes involving distributed supraspinal processing networks. Dogs with painful or unstable musculoskeletal conditions may demonstrate decreased willingness to perform previously routine movements, altered environmental engagement, frustration-associated behaviors, or changes in social interaction and exploratory activity. While these behavioral manifestations are variable and not universal across individuals, they parallel processes described in human chronic pain populations including fibromyalgia, osteoarthritis, and complex regional pain syndrome (3–7).

During rehabilitation, clinicians may observe improvements in movement confidence, behavioral engagement, and willingness to perform previously avoided tasks during recovery (9, 17–19). However, the mechanisms linking peripheral rehabilitation interventions with changes in affective-motivational and autonomic processing remain incompletely characterized. These observations support investigating broader interactions among peripheral tissue state, interoception, autonomic regulation, and central affective-motor processing networks within the conceptual framework proposed by the CMRA. Collectively, these observations provide the biological rationale for proposing the CMRA as a systems-level conceptual framework linking peripheral rehabilitation with distributed neurobehavioral adaptation.

### 1.3 The FoK–FIP theory: A conceptual framework linking mitochondrial function, interoception, and electromagnetic signaling

Recent theoretical developments have explored how cellular bioenergetics, interoception, and distributed physiological signaling processes may contribute to higher-order affective and cognitive phenomena. The Feelings of Knowing–Fundamental Interoceptive Patterns (FoK–FIP) theory proposes that mitochondrial activity could potentially contribute to organism-level interoceptive awareness through coordinated physiological and electromagnetic (EM) dynamics (44, 45). Within this conceptual framework, mitochondrial EM emissions are hypothesized not merely as metabolic byproducts, but as one possible component of broader signaling processes that may participate in how organisms sense and respond to internal physiological states (44, 45). The FoK–FIP framework further proposes that “feelings of knowing”—including pre-reflective awareness of bodily condition, internal physiological state, and implicit confidence in bodily perception—may emerge from distributed “fundamental interoceptive patterns” associated with coordinated mitochondrial and cellular activity across tissue networks (44, 45). Importantly, these concepts remain theoretical and are not currently established mechanistic explanations of consciousness or interoceptive processing.

Emerging evidence indicates that mitochondria generate ultra-weak electromagnetic phenomena associated with charge displacement, redox-related oscillatory activity, proton flux dynamics, and electron transport chain function (46–50). Related observations—including biophoton emission, oscillatory membrane potential fluctuations, and weak magnetic field perturbations—have prompted growing theoretical interest regarding potential relationships among bioenergetics, cellular signaling, network synchronization, and physiological organization across multiple spatial scales (51–55). At the same time, several theoretical frameworks in neuroscience and consciousness studies have proposed that endogenous EM field dynamics may contribute, to varying degrees, to large-scale neural coordination or integrative processing (56–59). However, these models remain debated, incompletely validated, and difficult to operationalize experimentally.

Building upon the FoK–FIP theory (44, 45), the present manuscript extends these comments by introducing the “resonome” as a conceptual term describing the proposed multiscale organization of endogenous EM-related physiological dynamics that may correlate with interoceptive processing, affective regulation, motor behavior, and broader network integration across biological systems. Within this framework, fascial and extracellular matrix networks are hypothesized to participate in distributed signaling and mechanobiological interactions that could potentially influence large-scale physiological coordination. Importantly, the resonome is introduced here as a theoretical construct and candidate organizing principle for interoceptive coherence rather than a validated biological architecture. The hypothesis that such organization contributes to rehabilitation-associated neurobehavioral adaptation remains experimentally testable and subject to future validation.

The CMRA framework further proposes that alterations in mitochondrial function, inflammatory burden, oxidative stress, autonomic dysregulation, or chronic nociceptive processing could potentially contribute to disrupted interoceptive and affective-motor integration through overlapping physiological mechanisms that may include bioenergetic, autonomic, inflammatory, and possibly EM-related processes. Conversely, rehabilitation interventions that improve tissue function, movement variability, autonomic regulation, and mitochondrial efficiency (60–65) may correlate with improvements in interoceptive processing, behavioral regulation, and motor adaptation. The specific biological mechanisms underlying these proposed relationships remain incompletely understood and require experimental validation; however, the framework generates testable predictions regarding how rehabilitation-associated changes in interoceptive organization may relate to behavioral, autonomic, and motor outcomes.

### 1.4 The fascial matrix as a potential distributed mechanobiological and bioelectrical interface

An additional component of the CMRA framework involves the hypothesis that fascia—the three-dimensional connective tissue continuum extending throughout the body—may participate in distributed mechanobiological and potentially bioelectrical signaling processes relevant to movement, interoception, autonomic regulation, and chronic pain adaptation (14, 15, 66–72). The fascial system includes dense irregular connective tissue, loose areolar connective tissue, and specialized fascial compartments that extend from superficial subcutaneous layers to deeper investing structures surrounding muscles, nerves, organs, and vascular networks, including the intramuscular perimysium and endomysium surrounding individual muscle fibers (66, 70). Contemporary fascial biology increasingly recognizes fascia as a dynamic and mechanosensitive tissue system rather than a purely passive structural material (14, 15). Several properties of fascial tissue have led investigators to explore whether fascia may contribute to broader physiological signaling and coordination processes, although many proposed mechanisms remain theoretical or incompletely validated.

#### 1.4.1 Piezoelectric properties

Collagen-containing connective tissues exhibit piezoelectric behavior, generating electrical potentials in response to mechanical deformation (71). This property has prompted interest in how movement, manual therapy, exercise, or tissue loading may influence local electromechanical environments and cellular signaling processes within connective tissues.

#### 1.4.2 Structural organization and liquid crystalline properties

The highly organized collagen architecture of fascial tissue has been proposed by some investigators to exhibit liquid crystalline characteristics that may support coordinated mechanical and electrochemical interactions across tissue networks (69). Although hypotheses involving large-scale electromagnetic signaling through fascial tissues remain speculative and experimentally difficult to validate, these structural properties may contribute to distributed mechanobiological organization and force transmission.

#### 1.4.3 Hydration layers and proton dynamics

Fascial tissues contain substantial water content and structured hydration layers adjacent to collagen interfaces (68). These interfacial water environments have been proposed to influence proton dynamics, charge distribution, and electrochemical interactions within biological tissues. However, the extent to which such properties contribute to physiologically meaningful large-scale signaling remains uncertain.

#### 1.4.4 Mechanoreceptor and sensory innervation

Fascial tissue contains dense populations of mechanoreceptors and free nerve endings capable of detecting mechanical, chemical, and thermal stimuli (73, 74). Through these sensory pathways, fascial tissue may contribute to proprioceptive input, autonomic regulation, nociceptive processing, and movement-related behavioral adaptation.

Nociceptive input arising from myofascial tissues can therefore engage established peripheral and central sensory pathways, linking local tissue events with spinal and supraspinal networks involved in sensory-discriminative and affective pain processing (7, 8, 73, 74). These established neuroanatomical pathways are distinct from the broader systems-level relationships hypothesized by the CMRA.

#### 1.4.5 Proposed mitochondrial–fascial interactions

Within the conceptual framework of the CMRA, mitochondria located within fascial fibroblasts, myofibroblasts, muscle-associated connective tissues, and adjacent cellular populations are hypothesized to participate in distributed bioenergetic and signaling interactions across tissue networks (44, 45). This hypothesis extends the FoK–FIP framework by proposing that interactions among mechanotransduction, mitochondrial function, inflammatory state, autonomic regulation, and tissue biomechanics may collectively influence interoceptive and affective-motor processing.

Under this interpretation, chronic pain, inflammation, altered tissue mechanics, or reduced movement variability could potentially contribute to disrupted physiological coordination through overlapping mechanisms involving nociceptive sensitization, autonomic dysregulation, altered tissue biomechanics, impaired mitochondrial function, and possibly broader bioelectrical interactions. Conversely, rehabilitation interventions such as therapeutic exercise, manual therapy, and movement-based loading may improve tissue perfusion, mechanical adaptability, autonomic regulation, and mitochondrial efficiency (60–65), potentially contributing to restoration of interoceptive coherence, improved movement confidence, and behavioral regulation.

Importantly, the proposed fascial-resonome interactions described here remain speculative and should not be interpreted as established mechanisms of long-distance electromagnetic communication or validated explanations of consciousness, cognition, or whole-body physiological coordination. The CMRA framework instead proposes that interactions among fascia, bioenergetics, interoception, chronic pain processing, and rehabilitation biology may contribute to organism-level adaptation through restoration of interoceptive coherence. While the biological mechanisms underlying these interactions remain incompletely understood, the framework generates experimentally testable predictions regarding rehabilitation-associated changes in behavior, autonomic regulation, and motor function.

### 1.5 Physiotherapy and multimodal rehabilitation as potential modulators of bioenergetic and interoceptive processes

Canine rehabilitation employs a broad range of multimodal interventions designed to improve tissue function, mobility, pain modulation, neuromuscular control, and overall quality of life (10, 11, 17, 18). These interventions may include:

- Manual therapy techniques such as stretching, passive range of motion (PROM), soft tissue mobilization, myofascial release, instrument-assisted soft tissue mobilization (IASTM), and joint mobilization (75–84).
- Therapeutic exercise approaches including controlled limb loading, proprioceptive training, balance and coordination exercises, neuromuscular strengthening, and aquatic therapy (85–92).
- Biophysical modalities such as extracorporeal shockwave therapy (ESWT), photobiomodulation (PBM), therapeutic ultrasound, neuromuscular electrical stimulation (NMES), transcutaneous electrical nerve stimulation (TENS), pulsed electromagnetic field therapy (PEMF), nuclear magnetic resonance therapy (MBST), cryotherapy, thermotherapy, dry needle acupuncture (DN), electroacupuncture (EA), and aqua-acupuncture with vitamin B12 (60–65, 85, 93–109)
- Regenerative medicine interventions including platelet-rich plasma (PRP), stem cell therapies, and placenta-derived biologic products (110–123).
- Pharmacologic and nutraceutical strategies including nonsteroidal anti-inflammatory drugs (NSAIDs), medications used in neuropathic pain management, omega-3 fatty acids, and glucosamine formulations (124–127).

Many of these interventions have documented effects on tissue perfusion, inflammation, mechanotransduction, mitochondrial metabolism, redox signaling, calcium-dependent pathways, neuromuscular activation, and autonomic regulation (16, 60–65, 128). For example, photobiomodulation, pulsed electromagnetic field therapy, extracorporeal shockwave therapy, and electroacupuncture have each been associated in experimental contexts with alterations in mitochondrial activity, ATP production, reactive oxygen species signaling, or markers of cellular bioenergetic adaptation. Similarly, manual therapies and movement-based rehabilitation approaches may influence fascial tissue mechanics, proprioceptive signaling, circulation, and autonomic responsiveness.

Within the conceptual framework of the CMRA, these overlapping physiological effects are hypothesized to interact with broader systems involved in interoception, affective regulation, motor behavior, and chronic pain adaptation. Rather than proposing that rehabilitation interventions directly “restore electromagnetic coherence,” the present framework proposes that improvements in tissue health, movement variability, autonomic balance, mitochondrial efficiency, and sensorimotor integration may contribute to restoration of interoceptive coherence through multiple interacting biological pathways, some of which may involve broader bioelectrical dynamics. Within the CMRA framework, restoration of interoceptive coherence is hypothesized to represent a candidate mediating process linking peripheral rehabilitation interventions to affective, autonomic, nociceptive, and motor adaptation.

Under this interpretation, rehabilitation-associated improvements in gait behavior, movement confidence, autonomic regulation, behavioral engagement, and pain-related function may reflect integrated changes across peripheral tissue state, inflammatory signaling, neuromuscular control, central sensitization, and distributed neurophysiological processing networks. The proposed relationships remain theoretical, non-exclusive, and incompletely validated. The CMRA is not presented as a definitive mechanistic explanation; rather, it is proposed as a testable systems-level hypothesis in which restoration of interoceptive coherence serves as a candidate mediating variable linking rehabilitation-associated physiological adaptation to functional recovery. The specific biological mechanisms underlying this process remain subject to experimental investigation.

### 1.6 Objective and structure of this hypothesis and theory article

The present manuscript introduces the Cingulate–Mitochondrial–Resonome Axis (CMRA) as a testable systems-level hypothesis proposing that rehabilitation-associated restoration of interoceptive coherence may serve as a candidate mediating process linking peripheral physiological adaptation to affective, autonomic, nociceptive, and motor change. Building upon concepts derived from the FoK–FIP framework and related literature involving mechanotransduction, autonomic regulation, fascia biology, and cingulate-mediated processing, the CMRA integrates these previously disconnected domains into a unified conceptual framework that generates experimentally testable predictions. While the specific biological mechanisms underlying resonome organization remain incompletely understood, the central hypothesis itself is presented as a falsifiable explanatory framework for future investigation.

To facilitate falsifiability and translational investigation, the manuscript proposes several clinically feasible multimodal outcome domains that could potentially be explored in future canine rehabilitation studies. These include:

1. Infrared thermography (IRT) of the medial caruncle as a potential indicator of autonomic activity and physiological arousal states (129–131).
2. Quantitative gait analysis as a measure of movement adaptation, motor behavior, and functional recovery (132).
3. Validated behavioral questionnaires assessing pain-related function, frustration-associated behavior, impulsivity, and quality-of-life related changes (133–136).

Importantly, these proposed outcome measures are not specific validations of the CMRA framework and may reflect multiple overlapping biological mechanisms, including altered biomechanics, reduced inflammation, changes in central sensitization, autonomic adaptation, improved conditioning, or other conventional rehabilitation effects.

The manuscript also outlines the conceptual basis for a future pilot rehabilitation application within a naturalistic veterinary clinical environment. This proposed setting is intended primarily to illustrate translational feasibility and multimodal outcome integration rather than to establish mechanistic proof of the CMRA framework.

The manuscript is structured as follows: Section 2 introduces the central hypothesis and associated predictions; Section 3 develops the broader theoretical framework underlying the CMRA across multiple organizational levels; Section 4 discusses translational implications for canine rehabilitation; Section 5 outlines proposed multimodal assessment approaches; Section 6 explores potential mechanistic interpretations linking rehabilitation interventions with affective-motor and physiological adaptation; Section 7 presents testable predictions and falsifiability considerations; Section 8 discusses limitations, alternative explanations, and future directions; and Section 9 provides concluding synthesis.

## 2 Core hypothesis and exploratory predictions

### 2.1 Statement of the core hypothesis

The central hypothesis of the CMRA is that rehabilitation acts, in part, by restoring the coherence and organization of interoceptive signaling across interacting physiological systems. Within this framework, restoration of interoceptive coherence is proposed as a candidate mediating process linking rehabilitation-associated changes in tissue biomechanics, inflammatory state, autonomic regulation, mitochondrial function, and sensorimotor activity to cingulate-mediated affective-motor processing and behavioral adaptation. Physiotherapy and multimodal rehabilitation interventions are therefore hypothesized to influence functional recovery not solely through local tissue repair, but also through organism-level reorganization of interoceptive processing.

Building upon the FoK–FIP framework, the CMRA further hypothesizes that endogenous electromagnetic (EM)-related physiological dynamics associated with mitochondrial activity may contribute, in part, to distributed interoceptive and neurophysiological organization across biological systems. The term “resonome” is used here as a conceptual descriptor for the proposed multiscale organization of interoceptive signaling dynamics and their integration across biological systems rather than as a validated anatomical or electrophysiological structure. Within this interpretation, fascial and connective tissue networks are hypothesized to participate in mechanobiological and potentially bioelectrical interactions that may contribute to coordination among peripheral tissue state, autonomic regulation, interoceptive processing, and motor behavior (14, 45–54, 137–139).

According to the CMRA framework, rehabilitation-associated improvements in tissue function, movement variability, autonomic regulation, and mitochondrial efficiency (16, 60–65, 128) could potentially correlate with measurable changes in gait behavior, movement confidence, behavioral engagement, pain-related function, and physiological stress regulation. In some individuals, improvements in frustration-associated behaviors, affective regulation, or motivational engagement may also occur. Within the CMRA framework, these outcomes are hypothesized to arise when rehabilitation-associated physiological adaptation improves the coherence and stability of interoceptive signaling, thereby altering how bodily state information is integrated within affective, autonomic, nociceptive, and motor networks. However, these proposed relationships are not assumed to be unique to EM-related mechanisms and may reflect multiple overlapping biological processes, including altered biomechanics, reduced inflammation, improved conditioning, central sensitization changes, or broader neurophysiological adaptation.

Importantly, the CMRA is not presented as a validated mechanistic explanation of consciousness, interoception, or large-scale electromagnetic coordination within biological systems. Rather, it is proposed as a falsifiable systems-level hypothesis whose central predictions can be experimentally tested while the specific biological mechanisms underlying resonome organization remain subject to ongoing investigation.

### 2.2 Exploratory predictions derived from the FoK–FIP and CMRA framework

The CMRA framework proposes that rehabilitation may influence chronic pain adaptation and functional recovery through interacting mechanisms involving tissue biomechanics, autonomic regulation, interoception, mitochondrial bioenergetics, and broader neurophysiological organization. Within this conceptual interpretation, changes in tissue state and sensorimotor processing could potentially correlate with alterations in affective-motor regulation, behavioral adaptation, and physiological stress responsiveness. The following exploratory predictions are intended as hypothesis-generating propositions rather than validated mechanistic conclusions.

#### 2.2.1 Prediction 1: rehabilitation may influence interoceptive and bioenergetic processes relevant to chronic pain adaptation

Rehabilitation interventions including manual therapy, therapeutic exercise, photobiomodulation, and related modalities may improve tissue mobility, mitochondrial metabolism, circulation, inflammatory regulation, and sensorimotor integration (16, 60–65, 128). Within the CMRA framework, these overlapping physiological changes are hypothesized to contribute to more stable interoceptive processing and improved behavioral adaptation during recovery from chronic musculoskeletal dysfunction.

#### 2.2.2 Prediction 2: distributed tissue and sensorimotor interactions May contribute to widespread functional changes

Because fascial, autonomic, sensorimotor, and behavioral systems are highly interconnected, localized rehabilitation interventions may sometimes correlate with broader changes in movement coordination, autonomic responsiveness, or pain-related behavior extending beyond the directly treated region. These effects may arise through multiple overlapping mechanisms including altered biomechanics, autonomic modulation, changes in central sensitization, movement variability, or distributed mechanobiological interactions.

#### 2.2.3 Prediction 3: improvements in tissue function and sensorimotor integration may correlate with changes in interoceptive processing

The CMRA framework predicts that reductions in nociceptive burden, improved movement quality, and enhanced tissue adaptability could potentially correlate with improved interoceptive accuracy and more stable affective-motor regulation. Within the conceptual language of the framework, such changes are described as improved organization of interoceptive and physiological signaling dynamics rather than as direct proof of electromagnetic coherence mechanisms.

#### 2.2.4 Prediction 4: rehabilitation may influence cingulate-mediated affective-motor processing

Because anterior and midcingulate networks participate in affective regulation, movement-related decision-making, autonomic processing, and pain adaptation (34–37, 45–56, 133, 137, 138, 140, 141), rehabilitation-associated improvements in interoceptive and sensorimotor processing may correlate with altered behavioral responses to pain, movement confidence, frustration-associated behavior, and motor planning during recovery.

#### 2.2.5 Prediction 5: multimodal outcome measures ay detect parallel changes across behavioral, autonomic, and functional domains

If rehabilitation influences distributed physiological and affective-motor systems as proposed, measurable changes may occur across multiple outcome domains simultaneously. Infrared thermography, quantitative gait analysis, and validated behavioral instruments may therefore provide complementary, although non-specific, indicators of rehabilitation-associated neurophysiological and behavioral adaptation.

Importantly, such findings would not uniquely validate the CMRA framework and could also reflect conventional rehabilitation mechanisms including improved biomechanics, reduced inflammation, altered conditioning, and changes in central sensitization.

#### 2.2.6 Prediction 6: functional improvements may occur despite persistent structural abnormalities

The framework predicts that clinically meaningful improvements in movement quality, behavioral engagement, and autonomic regulation may occur even when structural pathology remains detectable. This possibility is consistent with broader chronic pain literature demonstrating that functional outcomes are often influenced by central processing, behavioral adaptation, and affective state (26–33) in addition to static anatomical findings.

#### 2.2.7 Prediction 7: multimodal rehabilitation approaches may produce broader functional adaptation than isolated interventions

The CMRA framework proposes that multimodal rehabilitation approaches targeting movement variability, tissue mechanics, autonomic regulation, mitochondrial metabolism, and behavioral engagement may produce broader functional improvements than interventions directed toward isolated tissue pathology alone. The framework further predicts that treatment frequency, intensity, and consistency may influence behavioral, gait-related, and autonomic outcomes over time, although these relationships remain speculative and require formal experimental evaluation.

#### 2.3 Distinguishing predictions and falsification criteria

While several predictions generated by the CMRA framework overlap with observations that may also be explained by established models of chronic pain adaptation, central sensitization, predictive processing, or neuroplastic rehabilitation, two predictions are particularly useful for evaluating whether the broader systems-level relationships proposed by the CMRA are supported beyond predominantly local biomechanical explanations of rehabilitation outcomes.

##### 2.3.1 Prediction A: functional improvement may exceed structural improvement

The CMRA framework predicts that clinically meaningful improvements in gait, behavioral engagement, autonomic regulation, and affective-motor function may occur despite persistent structural pathology visible on diagnostic imaging. Within the framework, this dissociation is hypothesized to occur when rehabilitation improves the coherence and organization of interoceptive signaling even though anatomical abnormalities remain present.

Potential Falsification: If functional recovery consistently requires proportional structural resolution, and meaningful functional improvement never occurs in the presence of persistent pathology, this prediction would not be supported.

##### 2.3.2 Prediction B: non-local effects May exceed local biomechanical expectations

The CMRA framework predicts that interventions directed at one anatomical region may occasionally correlate with improvements in distant functional domains that exceed what would be expected from local biomechanical mechanisms alone. Such observations could include changes in autonomic regulation, behavioral engagement, movement confidence, or broader movement coordination following localized rehabilitation interventions.

Potential Falsification: If rehabilitation effects remain strictly localized and no reproducible non-local improvements are observed beyond established biomechanical explanations, this prediction would not be supported.

#### 2.4 Summary

Together, these exploratory predictions outline a conceptual pathway through which rehabilitation-associated changes in tissue state, movement behavior, autonomic regulation, and interoceptive processing could potentially interact across multiple biological levels. Rather than serving as proof of electromagnetic coordination mechanisms, these predictions are intended to provide a structured and experimentally approachable framework for investigating broader interactions among chronic pain, rehabilitation, interoception, and affective-motor adaptation.

## 3 Theoretical foundations of the cingulate–mitochondrial–resonome axis

### 3.1 Mitochondrial bioenergetics, oscillatory physiology, and proposed electromagnetic contributions to interoceptive processing

Mitochondria exhibit diverse electrochemical and biophysical properties—including oscillatory membrane potentials, proton flux dynamics, redox cycling, calcium signaling interactions, and electron transport chain activity—that contribute to cellular bioenergetics and physiological regulation (142–162). These processes are associated with measurable ultra-weak electromagnetic phenomena, including fluctuating membrane potentials, charge displacement, magnetic perturbations, and biophoton emission (75–78, 93, 163–169). Although experimentally detectable, the broader physiological significance of these phenomena at tissue or organismal scales remains incompletely understood.

Within the CMRA and FoK–FIP framework, mitochondrial oscillatory dynamics are hypothesized to contribute, in part, to physiological processes relevant to interoception, autonomic regulation, affective processing, and movement adaptation.

Interest in potential relationships among mitochondrial dynamics, mechanotransduction, and systems-level physiological organization is supported by observations involving coordinated metabolic oscillations, redox-sensitive signaling interactions, cytoskeletal coupling, calcium-dependent synchronization processes, and reported biological sensitivity to weak electromagnetic influences (75–78, 93, 163–169). However, the extent to which these phenomena contribute to large-scale physiological integration or interoceptive processing remains uncertain.

The CMRA framework further proposes that connective tissue organization, hydration dynamics, mechanobiological interactions, autonomic regulation, inflammation, and mitochondrial metabolism may collectively influence how physiological signals are distributed and integrated during rehabilitation. Rather than asserting validated long-distance electromagnetic signaling through fascial tissues, the framework explores whether overlapping biomechanical and physiological processes may contribute to broader affective-motor and interoceptive adaptation.

Many rehabilitation interventions—including therapeutic exercise, photobiomodulation, manual therapy, and electroacupuncture—have documented effects on mitochondrial metabolism, inflammatory signaling, tissue mechanics, circulation, and autonomic responsiveness (85–87, 94–98, 170, 171). Within the CMRA framework, these physiological effects are hypothesized to interact with broader processes involved in pain adaptation, movement behavior, and interoceptive regulation. However, proposed relationships involving electromagnetic organizational processes remain speculative and require substantial experimental validation.

Accordingly, the CMRA does not propose validated organism-level electromagnetic signaling hierarchies or direct restoration of “electromagnetic coherence.” Rather, the framework suggests that rehabilitation-associated improvements in tissue function, movement variability, mitochondrial physiology, and autonomic regulation may correlate with broader changes in behavioral adaptation and affective-motor processing through multiple interacting mechanisms.

#### 3.2 Fascial architecture, biotensegrity, and distributed mechanobiological organization

##### 3.2.1 Fascial organization and biotensegrity concepts

The fascial system represents a body-wide connective tissue continuum that some investigators describe using principles related to biotensegrity—a biological adaptation of tensegrity architecture in which tension-bearing and compression-resistant elements interact to support mechanical stability, force distribution, and adaptive movement behavior (172, 173). Within this perspective, fascia may contribute to large-scale mechanical integration across musculoskeletal and visceral systems while also participating in proprioceptive, autonomic, and interoceptive processes relevant to chronic pain adaptation and rehabilitation.

Several properties of fascial organization have motivated theoretical interest regarding broader physiological coordination processes (172, 173).

##### 3.2.1.1 Distributed mechanical force transmission

Unlike discrete anatomical pathways such as nerves or vascular structures, fascial tissues distribute mechanical forces across interconnected tissue networks (174, 175). Consequently, local movement, tissue loading, or manual therapy interventions may influence adjacent or functionally related regions through conventional biomechanical and neuromuscular mechanisms. Some investigators have proposed that such distributed interactions may also contribute to broader interoceptive and physiological integration, although these relationships remain incompletely understood.

##### 3.2.1.2 Baseline tissue tension and mechanical adaptation

Fascial tissues exist under varying degrees of pre-tension that help maintain structural relationships among muscles, connective tissues, organs, and skeletal elements (176). These mechanical properties influence tissue stiffness, force transmission, elastic recoil, and movement coordination (176). Within the CMRA framework, such tissue organization is hypothesized to potentially influence local mechanobiological, electromechanical, and bioenergetic interactions, although evidence supporting large-scale electromagnetic signaling through fascial tissues remains limited.

##### 3.2.1.3 Hierarchical structural organization

Fascial tissues exhibit hierarchical organization across multiple spatial scales, ranging from collagen fibrils and extracellular matrix structure to larger fascial planes and regional connective tissue networks (177, 178). This multiscale architecture may support coordinated mechanical and physiological interactions during movement and rehabilitation adaptation. Within the CMRA framework, these organizational features are hypothesized to potentially contribute to distributed interoceptive and affective-motor processes through overlapping biomechanical, autonomic, and bioenergetic interactions. However, proposed relationships involving large-scale electromagnetic coordination remain theoretical and experimentally unresolved.

#### 3.2.2 Fascial cellular networks, mechanotransduction, and bioenergetic interactions

Fascial tissues contain metabolically active fibroblasts, myofibroblasts, immune-associated cells, vascular elements, and sensory interfaces that participate in extracellular matrix maintenance, mechanotransduction, inflammatory regulation, and tissue adaptation (178). These cellular systems are increasingly recognized as biologically dynamic rather than passive structural components and may contribute to physiological responses associated with movement, chronic pain, and rehabilitation (178).

##### 3.2.2.1 Mechanotransduction and mitochondrial interactions

Fascial fibroblasts respond to mechanical loading and tissue deformation through alterations in cytoskeletal organization, cellular signaling, mitochondrial metabolism, and ATP production (179, 180). These mechanobiological interactions provide one pathway through which movement, manual therapy, and rehabilitation-related tissue loading may influence local physiological adaptation. Within the CMRA framework, such interactions are hypothesized to potentially contribute to broader interoceptive and affective-motor regulation through overlapping biomechanical, autonomic, inflammatory, and bioenergetic processes.

##### 3.2.2.2 Extracellular matrix remodeling and tissue adaptation

Fascial cells continuously remodel extracellular matrix components including collagen, elastin, and ground substance in response to mechanical loading, inflammation, and metabolic state (181–184). Chronic pain, immobility, and persistent inflammatory states are associated with altered tissue mechanics, increased collagen cross-linking, reduced hydration, and impaired connective tissue adaptability (185, 186). Rehabilitation interventions may improve tissue mobility, hydration, circulation, and mechanical responsiveness through established mechanobiological processes. Within the CMRA framework, these tissue-level changes are hypothesized to potentially influence broader physiological adaptation through effects on interoceptive processing, autonomic regulation, and affective-motor integration.

##### 3.2.2.3 Distributed sensory and physiological responsiveness

Emerging evidence suggests that fascial cellular systems respond to mechanical deformation, inflammatory mediators, autonomic signaling, pH variation, and other local physiological conditions (187, 188). These properties support the view that fascial tissues participate in distributed sensory and adaptive processes relevant to movement behavior, nociceptive regulation, and autonomic adaptation. Proposed relationships involving large-scale electromagnetic coordination or consciousness-related signaling, however, remain theoretical and experimentally unresolved.

#### 3.2.3 Fascial structural organization, hydration dynamics, and proposed electromechanical interactions

The molecular organization of collagen-rich connective tissues has been described by some investigators as exhibiting semi-ordered or liquid crystalline structural properties that may support mechanical adaptability and coordinated electrochemical interactions within biological tissues (189, 190). These concepts remain an active area of theoretical and experimental investigation in relation to mechanotransduction, hydration dynamics, tissue organization, and physiological signaling (189, 190).

#### 3.2.3.1 Structural organization and coordinated interactions

Some theoretical models propose that semi-ordered collagen architectures may facilitate coordinated molecular and electromechanical interactions across connective tissue systems (191, 192). Within the CMRA framework, such organizational properties are hypothesized to potentially influence how local physiological signals associated with tissue loading, mitochondrial activity, hydration dynamics, and cellular signaling interact across interconnected tissue environments. However, direct evidence supporting large-scale electromagnetic propagation through fascial tissues remains limited.

#### 3.2.3.2 Frequency-dependent tissue behavior

Connective tissues exhibit frequency-dependent mechanical and viscoelastic behavior under varying loading conditions (193). Some investigators have proposed that tissue organization and hydration state may influence local electromechanical interactions or responsiveness to externally applied biophysical modalities. Within the CMRA framework, these ideas are explored as possible contributors to variable physiological responses observed during rehabilitation interventions, although proposed electromagnetic mechanisms remain theoretical and incompletely validated.

#### 3.2.3.3 Hydration state and tissue adaptability

Hydration substantially influences connective tissue mechanics, viscoelastic behavior, and extracellular matrix organization (194, 195). Dehydration and impaired tissue mobility may alter tissue adaptability, whereas movement, manual therapy, and rehabilitation interventions can influence fluid distribution and connective tissue dynamics through established biomechanical processes. Within the CMRA framework, hydration-dependent changes in tissue organization are hypothesized to potentially contribute to broader physiological adaptation, although these relationships remain exploratory and should not be interpreted as established mechanisms of large-scale electromagnetic signaling.

#### 3.2.4 Hydration dynamics, interfacial water, and proposed electrochemical interactions

The surfaces of collagen-rich connective tissues are associated with structured hydration environments that differ from bulk water in their physical and electrochemical behavior (196). Some investigators have proposed that these interfacial water layers may influence local charge distribution, proton dynamics, tissue conductivity, and electromechanical interactions within biological systems (196). However, the physiological significance of these properties at larger tissue or organismal scales remains incompletely understood.

##### 3.2.4.1 Proton dynamics and tissue conductivity

Structured hydration layers adjacent to collagen interfaces have been hypothesized to facilitate proton exchange and localized electrochemical interactions within connective tissues (197, 198). Within the CMRA framework, such properties are explored as possible contributors to local electromechanical responsiveness and tissue-level physiological adaptation. Direct evidence supporting large-scale biological communication networks mediated through fascial proton conduction, however, remains limited.

##### 3.2.4.2 Electromagnetic energy absorption and bioenergetic interactions

Interfacial water systems may interact with externally applied electromagnetic energy, including infrared wavelengths relevant to photobiomodulation research (199). Some theoretical models propose that hydration-dependent electrochemical dynamics could contribute to local or regional physiological responses to biophysical interventions. Within the CMRA framework, these ideas are explored as speculative hypotheses regarding interactions among hydration state, mitochondrial metabolism, tissue responsiveness, and rehabilitation-associated adaptation rather than validated mechanisms explaining systemic treatment effects.

##### 3.2.4.3 Mechanical responsiveness and tissue state

Mechanical loading, compression, and tissue stretch influence hydration distribution, extracellular matrix organization, and connective tissue mechanics (200, 201). Rehabilitation interventions such as movement therapy, manual therapy, and myofascial techniques may therefore alter local tissue dynamics through established biomechanical and physiological processes. Within broader CMRA interpretations, hydration-dependent changes in tissue organization are hypothesized to potentially influence interoceptive and autonomic processes, although these relationships remain theoretical and experimentally unresolved.

### 3.3 The resonome as a conceptual multiscale framework for physiological and interoceptive organization

Building upon the FoK–FIP framework and broader concepts involving mechanotransduction, bioenergetics, interoception, and distributed physiological organization (202–208), the CMRA introduces the “resonome” as a conceptual descriptor describing how interacting biological processes may organize across multiple spatial and functional scales. Rather than representing a validated electromagnetic hierarchy, the resonome is presented as a speculative interpretive framework exploring possible relationships among mitochondrial dynamics, connective tissue organization, autonomic regulation, sensorimotor processing, and affective-motor integration. Within this framework, mitochondrial metabolism, redox cycling, calcium signaling, and oscillatory physiological activity contribute to cellular bioenergetics and local physiological regulation (202, 203), while fascial and connective tissue systems participate in mechanotransduction, extracellular matrix remodeling, inflammatory signaling, metabolic adaptation, and intercellular communication (204). Functional interactions among fascia, musculature, hydration dynamics, autonomic innervation, proprioceptive processing, and sensorimotor adaptation influence biomechanical and physiological responses during movement and rehabilitation (205–208).

Cingulate and related cortical networks participate in nociceptive, interoceptive, affective, and behavioral processes relevant to chronic pain and rehabilitation (209–212). Together, these interacting systems provide a conceptual framework for exploring how rehabilitation-associated physiological adaptation may influence interoceptive regulation, movement behavior, autonomic regulation, and affective-motor processing across multiple biological scales. Rather than proposing a validated model of large-scale electromagnetic coordination, the CMRA offers an exploratory interdisciplinary interpretation of how distributed physiological processes may contribute to chronic pain adaptation and behavioral recovery. Proposed electromagnetic contributions to these interactions remain theoretical and require substantial experimental validation. Figure 1.

**Figure 1:**
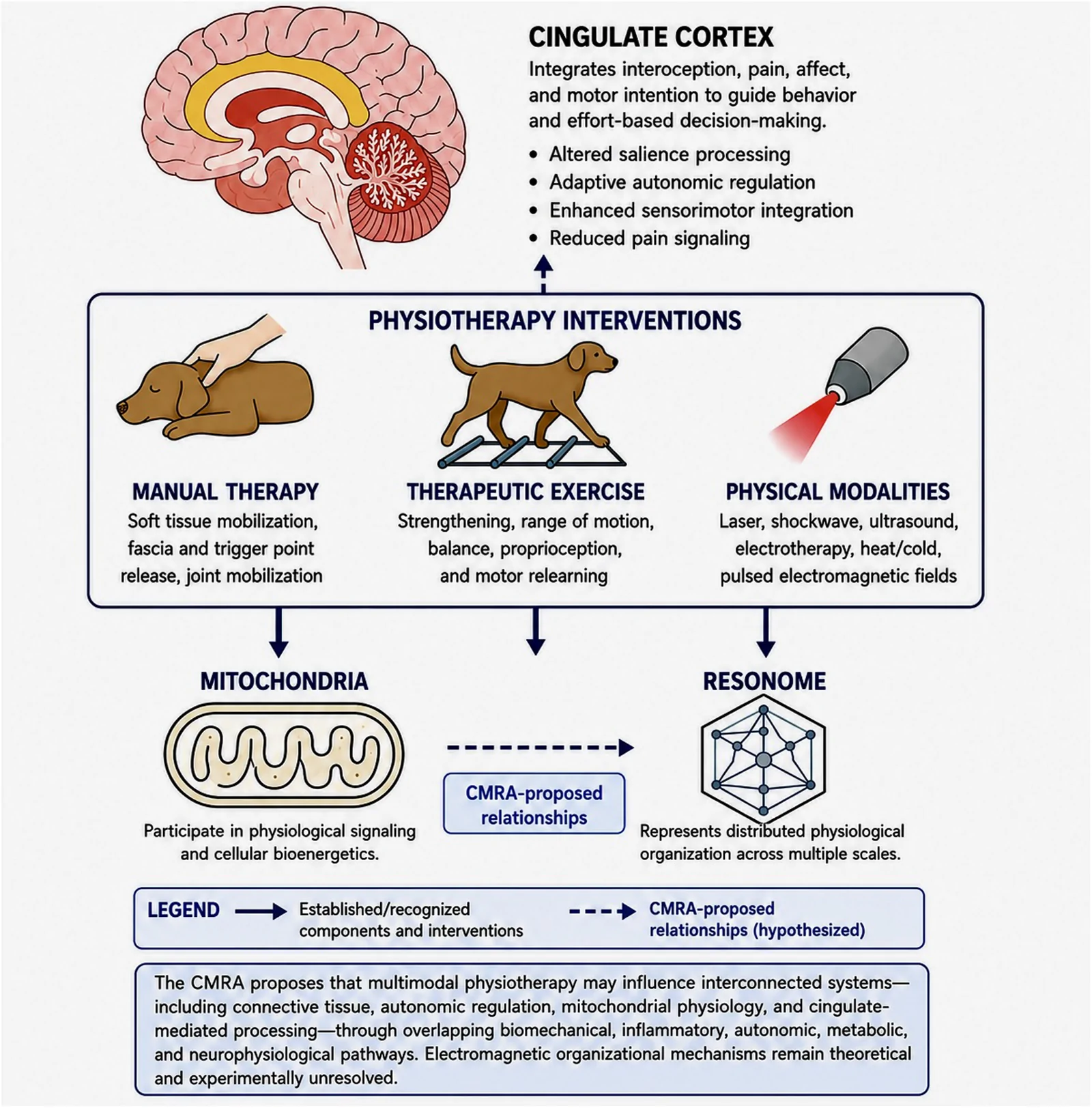
Conceptual overview of the cingulate–mitochondrial–resonome axis (CMRA). This schematic illustrates the CMRA as an exploratory conceptual framework examining potential interactions among rehabilitation interventions, mitochondrial physiology, connective tissue organization, autonomic regulation, interoceptive processing, and cingulate-mediated affective-motor adaptation. Arrows depicting CMRA-specific relationships represent hypothesized associations rather than established causal pathways. The framework proposes that multimodal rehabilitation may influence distributed physiological systems relevant to chronic pain adaptation and functional recovery through overlapping biomechanical, inflammatory, autonomic, metabolic, and neurophysiological processes. Proposed electromagnetic organizational mechanisms remain theoretical and experimentally unresolved.

### 3.4 Cingulate Cortex integration of interoceptive, affective, and sensorimotor processing

The cingulate cortex occupies a central neuroanatomical position within networks involved in interoception, nociceptive evaluation, autonomic regulation, affective processing, and movement-related behavioral adaptation (209–212). Within the CMRA framework, the cingulate cortex is hypothesized to integrate physiological information arising from interactions among peripheral tissue state, autonomic regulation, movement behavior, and broader interoceptive processes rather than serving as a direct interpreter of discrete electromagnetic signals.

#### 3.4.1 Anterior cingulate cortex (ACC)

The anterior cingulate cortex (ACC) participates in affective pain processing, motivational salience attribution, autonomic regulation, conflict monitoring, and behavioral responses to perceived threat or discomfort (213–218). Functional neuroimaging studies demonstrate that ACC activity correlates more strongly with the emotional and motivational dimensions of pain than with nociceptive intensity alone (219).

Within the CMRA framework, the ACC is interpreted as integrating interoceptive, autonomic, nociceptive, and contextual information relevant to behavioral prioritization and affective-motivational regulation during chronic pain states. Rehabilitation-associated changes in movement behavior, autonomic regulation, inflammatory burden, and interoceptive processing may therefore influence ACC-mediated behavioral adaptation through multiple interacting physiological mechanisms. Proposed contributions involving broader bioelectrical or electromagnetic dynamics remain speculative and experimentally unresolved.

#### 3.4.2 Midcingulate cortex (MCC)

The midcingulate cortex (MCC) contributes to action selection, movement planning, effort allocation, motor adaptation, and behavioral responses under conditions of pain, uncertainty, or anticipated effort (41, 220–227). Electrophysiological recordings in animal models demonstrate that MCC activity reflects movement-related decision-making, reward evaluation, action cost estimation, and adaptive behavioral control (228, 229).

Within the CMRA framework, the MCC is hypothesized to participate in integrating sensorimotor, interoceptive, autonomic, and affective information relevant to movement confidence, gait adaptation, and motor planning during rehabilitation. The framework proposes that distributed physiological changes associated with chronic pain and rehabilitation may influence MCC-mediated behavioral adaptation through interacting biomechanical, autonomic, inflammatory, and neurophysiological pathways.

#### 3.4.3 Posterior cingulate cortex (PCC) and default mode integration

The posterior cingulate cortex (PCC) participates in self-referential processing, internal state monitoring, memory integration, and aspects of interoceptive attention allocation (230–235). Although less emphasized within the current CMRA framework, PCC-related networks may contribute to how chronic pain states influence behavioral expectation, bodily self-monitoring, and adaptive prediction regarding future movement or discomfort.

Together, anterior, midcingulate, and posterior cingulate networks support dynamic integration of affective processing, autonomic regulation, interoception, nociceptive evaluation, and movement-related behavioral adaptation (41, 209–235). Within the CMRA framework, rehabilitation-associated physiological adaptation involving peripheral tissue state, movement variability, inflammatory signaling, autonomic regulation, and interoceptive processing is hypothesized to potentially influence these distributed systems through multiple overlapping physiological mechanisms. Importantly, the CMRA does not propose that conscious awareness, affective experience, or motor behavior arise directly from discrete electromagnetic signaling pathways. Rather, the framework explores whether interactions among biomechanics, bioenergetics, autonomic regulation, interoception, and neurophysiological organization may contribute to chronic pain adaptation and rehabilitation-related behavioral change across multiple biological scales.

## 4 Application to canine rehabilitation: mechanisms and clinical context

### 4.1 Relevance of cingulate-mediated processing in dogs with chronic musculoskeletal disease

Many dogs with chronic orthopedic and neuromuscular disease exhibit behavioral and movement-related changes associated with chronic pain and musculoskeletal dysfunction. Reported manifestations include reduced activity, altered movement behavior, pain-related behavioral changes, and changes in mobility and functional engagement (3, 5, 9, 138, 140, 141).

These observations parallel findings in human chronic pain literature demonstrating interactions among chronic pain, cingulate-related processing, affective adaptation, autonomic regulation, and movement-related behavior. Chronic pain is associated with structural and functional brain plasticity (236, 237), while cingulate regions participate in affective pain processing, autonomic regulation, effort-related decision-making, and motor-related behavioral control (7, 22, 41, 216, 219, 224, 238).

Comparative neuroanatomical and functional evidence supports meaningful similarities between canine and human brain organization relevant to the present framework. Canine neuroimaging demonstrates functional homology in large-scale brain networks, including the default mode network (239), while canine neuroanatomical mapping provides a basis for examining corresponding cortical structures (240). Dogs may therefore provide a useful translational model for investigating how chronic musculoskeletal disease influences interactions among movement behavior, autonomic regulation, interoceptive processing, affective adaptation, and rehabilitation-associated physiological change. Within the conceptual framework of the CMRA, these observations are interpreted as potentially reflecting interactions among peripheral tissue state, sensorimotor processing, autonomic regulation, and cingulate-mediated affective-motor integration. These interpretations remain theoretical and should not be interpreted as direct evidence for large-scale electromagnetic coordination mechanisms.

### 4.2 Rehabilitation modalities, bioenergetic processes, and interoceptive adaptation

#### 4.2.1 Manual therapy and myofascial interventions

Stretching, passive range of motion, soft tissue mobilization, myofascial release, and instrument-assisted soft tissue mobilization mechanically engage connective tissue and musculofascial systems (80). These interventions may influence circulation, tissue mobility, hydration dynamics, mechanotransduction, autonomic responsiveness, and inflammatory signaling (81–88, 205). Within the CMRA framework, such physiological changes are hypothesized to potentially contribute to interoceptive processing, movement confidence, and affective-motor adaptation during rehabilitation.

#### 4.2.2 Therapeutic exercise

Controlled limb loading, proprioceptive training, balance exercises, and neuromuscular strengthening influence mitochondrial metabolism, movement variability, sensorimotor integration, and tissue adaptation through established physiological pathways including PGC-1*α*-associated mitochondrial biogenesis and neuromuscular remodeling (89–92, 114, 241). Exercise may also support proprioceptive accuracy, autonomic regulation, behavioral confidence, and movement-related adaptation during chronic pain rehabilitation.

#### 4.2.3 Biophysical modalities

Photobiomodulation (PBM), pulsed electromagnetic field therapy (PEMF), extracorporeal shockwave therapy (ESWT), therapeutic ultrasound, and related modalities influence mitochondrial metabolism, inflammatory signaling, tissue perfusion, mechanotransduction, and cellular stress responses (100–110). These interventions may contribute to pain modulation, tissue repair, autonomic responsiveness, and movement-related adaptation through multiple overlapping physiological mechanisms. Within the CMRA framework, proposed electromagnetic contributions to these effects remain theoretical and incompletely validated.

#### 4.2.4 Acupuncture and electroacupuncture

Acupuncture and electroacupuncture involve mechanical and electrical stimulation at sites frequently associated with fascial planes, neurovascular bundles, and connective tissue interfaces (111–113). Experimental evidence suggests these interventions may influence peripheral nerve activity, descending pain modulation pathways, autonomic regulation, inflammatory signaling, and sensorimotor processing. Within the CMRA framework, these effects are interpreted as potentially involving interactions among connective tissue mechanics, interoceptive processing, autonomic adaptation, and broader physiological regulation.

#### 4.2.5 Regenerative medicine and placenta-derived biologics

Placenta-derived biologic products, platelet-rich plasma (PRP), stem cell therapies, and related regenerative approaches contain bioactive molecules capable of influencing inflammation, extracellular matrix remodeling, angiogenesis, tissue repair, and cellular metabolism Springer et al., 2018 (114); Kan et al., 2023; Ip et al., 2017 (116); Steen & Parnell, 2020 (117); Badylak & Gilbert, 2008 (118); Swinehart & Badylak, 2016 (119); Fraser et al., 1997 (120); Stern, 2008 (121); Nair et al., 2019 (122); Ozawa et al., 2004 (123); Kuffler, 2014; Serhan et al., 2015; Dalli & Serhan, 2020; Bang et al., 2014 (124); Finnerup et al., 2015 (125); Stillwell & Wassall, 2003 (126); Henrotin et al., 2012 (127). These interventions may support tissue healing, fascial mobility, mitochondrial function, and local physiological adaptation through multiple interacting biological pathways. Within the CMRA framework, regenerative therapies are hypothesized to potentially influence interoceptive processing and affective-motor adaptation indirectly through improvements in tissue state, inflammatory regulation, autonomic regulation, and movement-related function.

#### 4.2.6 Pharmacologic and nutraceutical interventions

Pharmacologic and nutraceutical interventions including NSAIDs, corticosteroids, gabapentin, omega-3 fatty acids, glucosamine-chondroitin formulations, and related supportive therapies influence inflammatory signaling, nociceptive processing, mitochondrial stress responses, and tissue metabolism through established physiological mechanisms. These interventions may contribute to improved movement behavior, pain modulation, autonomic adaptation, and rehabilitation tolerance during chronic musculoskeletal disease management (129–131, 242). Within the CMRA framework, such effects are interpreted as potentially contributing to broader interoceptive coherence and affective-motor adaptation through overlapping physiological processes.

### 4.3 The clinical rehabilitation setting as an ideal test environment

The proposed pilot study leverages a naturalistic canine rehabilitation clinic environment in which dogs present with diverse musculoskeletal and neurologic conditions including osteoarthritis, cranial cruciate ligament disease, iliopsoas and myofascial pain syndromes, tendinopathies, lumbosacral disease, intervertebral disc disease (IVDD), post-operative orthopedic recovery, and generalized movement dysfunction. This clinical heterogeneity provides a potentially useful translational setting for exploring the CMRA framework, as different orthopedic, neurologic, and myofascial conditions may involve varying combinations of nociceptive burden, altered movement behavior, autonomic dysregulation, proprioceptive dysfunction, inflammatory signaling, and compensatory motor strategies.

Within this setting, multimodal rehabilitation outcomes across behavioral, autonomic, and biomechanical domains could be evaluated for potential convergent patterns relevant to chronic pain adaptation and affective-motor regulation. Such findings would not constitute direct validation of the CMRA framework or proposed electromagnetic mechanisms, but may help determine whether broader systems-level relationships among rehabilitation, movement behavior, autonomic regulation, and interoceptive processing warrant further investigation.

The lead investigator's clinical training as a Doctor of Veterinary Medicine (DVM), Certified Canine Rehabilitation Practitioner (CCRP), Certified Veterinary Acupuncturist (CVA), and Certified Small Animal Myofascial Practitioner (CSMP) supports consistent implementation of multimodal rehabilitation interventions and integrated assessment across orthopedic, neurologic, behavioral, and movement-related domains. This multidisciplinary background facilitates detailed gait analysis, palpatory assessment of tissue mobility and discomfort, individualized rehabilitation planning, and longitudinal monitoring of functional and behavioral adaptation during treatment. Within the proposed pilot framework, these observations may provide exploratory insight into how rehabilitation-associated physiological adaptation and behavioral change interact across multiple systems relevant to chronic pain adaptation and recovery.

### 4.4 Proposed statistical methods

As this is a pilot study, we will prioritize feasibility assessment, descriptive analyses, and estimation of effect sizes rather than formal hypothesis testing given the limited sample size. Feasibility metrics will include the number of patients meeting inclusion criteria, the ability to collect usable longitudinal data within a defined timeframe (e.g., one year), and successful follow-up completion. These metrics will be summarized descriptively through enrollment rates, data completeness percentages, and retention/attrition rates.

The target sample size is up to 12 canine participants, selected based on anticipated recruitment feasibility, case availability, and the time and resource requirements associated with longitudinal follow-up in a pilot clinical setting rather than statistical power considerations. The study will be considered feasible if at least 10 of 12 targeted participants (≥83%) are successfully enrolled, complete baseline and endpoint data are obtained for at least 80% of enrolled participants, and at least 75% of participants complete the final follow-up assessment.

Means and standard deviations, medians and interquartile ranges, and frequencies and percentages will be used to characterize the sample and outcome measures. Point estimates, 95% confidence intervals, and effect size estimates (e.g., Cohen's d for continuous outcomes; risk ratios and odds ratios for binary outcomes where applicable) will be reported to estimate variability and inform future study design considerations.

Graphical methods will be used to visualize individual participant trajectories across the treatment period, including spaghetti plots demonstrating longitudinal individual-level changes with overlaid group-averaged smoothed curves to examine overall response patterns. Scatter plots with best-fit lines will also be used descriptively to explore associations among continuous variables and outcome measures. These visualizations are intended to assess heterogeneity in treatment-associated responses and identify potential moderators or correlates for future investigation.

To further explore treatment-associated changes, we will report descriptive summaries, point estimates, confidence intervals, and effect size estimates for primary and secondary outcomes when appropriate, including the number of dogs demonstrating favorable change between baseline and follow-up assessments. Non-parametric analyses (Wilcoxon signed-rank tests for paired comparisons and Spearman correlations for exploratory associations) will be used to examine potential relationships among infrared thermography (IRT), gait analysis, behavioral measures, and treatment variables. Given the small sample size, statistical power will be limited, and *p*-values will be interpreted cautiously as exploratory indicators rather than confirmatory evidence.

## 5 Multimodal assessment battery

### 5.1 Infrared thermography as an exploratory autonomic measure

#### 5.1.1 Rationale

The medial caruncle contains dense vascular structures influenced by autonomic regulation and sympathetic-parasympathetic balance (129). Previous work suggests that infrared thermography (IRT) of the periocular region may reflect changes in physiological arousal, stress responsiveness, and autonomic state under standardized conditions. Because anterior and midcingulate networks participate in autonomic regulation and affective processing (216), rehabilitation-associated changes in pain burden, movement confidence, stress responsiveness, and behavioral adaptation could potentially correlate with alterations in medial caruncle temperature patterns.

Within the CMRA framework, IRT is interpreted as a non-specific physiological measure that may reflect broader autonomic regulation and interoceptive adaptation during rehabilitation rather than as a direct measure of electromagnetic organization or cingulate activity.

#### 5.1.2 Protocol

High-resolution infrared thermography will be used to measure bilateral medial caruncle temperature under standardized environmental and handling conditions (131). Pre- and post-treatment images will be analyzed using blinded region-of-interest (ROI) methods to assess longitudinal changes over time.

#### 5.1.3 Exploratory outcomes

Potential exploratory findings may include changes in mean caruncle temperature, reduction in left-right asymmetry, stabilization of temperature variability across timepoints, and possible associations with behavioral, gait, functional, or autonomic outcome measures.

#### 5.1.4 Falsification considerations

Findings that would argue against proposed CMRA-related interpretations include:

- absence of any consistent association between IRT changes and clinical or behavioral improvement,
- highly inconsistent or random temperature fluctuations across timepoints,
- temperature changes occurring independently of functional or behavioral adaptation,
- or inability to detect reproducible patterns under standardized assessment conditions.

### 5.2 Quantitative gait mapping and motor adaptation

#### 5.2.1 Rationale

The midcingulate cortex (MCC) participates in movement planning, action selection, effort allocation, motor adaptation, and behavioral responses to pain and uncertainty (223–225). Chronic musculoskeletal disease can alter gait patterns through interactions among nociceptive burden, proprioceptive disruption, compensatory movement strategies, autonomic state, and learned behavioral adaptation. Rehabilitation-associated improvements in movement confidence, tissue function, sensorimotor integration, and pain modulation may therefore correlate with measurable changes in gait behavior and loading symmetry over time.

Within the CMRA framework, quantitative gait analysis is interpreted as an exploratory functional measure reflecting integrated motor and behavioral adaptation rather than as a direct measure of electromagnetic signaling or cingulate activity.

#### 5.2.2 Protocol

The GaitFour pressure-sensitive walkway system will be used to capture primary spatiotemporal gait parameters, including stride length, stride time, stance time, swing time, and pressure-related measures such as peak pressure and pressure–time integral (impulse) during ambulation across the walkway (132). Higher-order metrics including symmetry indices and coefficients of variation for temporal and spatial parameters will subsequently be calculated through post-processing analysis.

#### 5.2.3 Exploratory outcomes

Potential exploratory findings may include improved loading symmetry, increased peak pressure and pressure–time integral in previously underloaded limbs, reduced coefficients of variation for stride length and temporal gait measures, normalization of stance-to-swing ratios, and progressive stabilization of gait variability over time. Temporal relationships among biomechanical, behavioral, and autonomic measures may also be explored descriptively.

#### 5.2.4 Falsification considerations

Findings that would argue against proposed CMRA-related interpretations include:

- gait improvements occurring independently of any detectable behavioral or autonomic changes,
- absence of reproducible longitudinal gait adaptation patterns,
- minimal change in gait parameters despite extensive rehabilitation intervention,
- or no observable differences between multimodal rehabilitation approaches and more limited treatment strategies.

Importantly, observed gait improvements alone would not constitute validation of the CMRA framework, as such changes may arise through multiple conventional rehabilitation mechanisms including pain reduction, improved conditioning, altered biomechanics, or enhanced neuromuscular coordination.

### 5.3 Validated behavioral questionnaires and affective-motivational adaptation

#### 5.3.1 Rationale

Anterior cingulate and related limbic networks participate in affective pain processing, motivational salience, autonomic regulation, frustration-related behavior, and behavioral adaptation during chronic pain states (7, 22, 31, 216). Chronic musculoskeletal disease may therefore influence not only movement behavior but also social engagement, stress responsiveness, behavioral regulation, and activity-related confidence in some individuals.

Within the CMRA framework, rehabilitation-associated improvements in pain burden, movement behavior, autonomic regulation, and interoceptive processing are hypothesized to potentially correlate with changes in affective-motivational measures over time. Importantly, these behavioral outcomes are interpreted as non-specific indicators of broader rehabilitation-associated adaptation rather than as direct measures of electromagnetic organization or cingulate function.

#### 5.3.2 Instruments

Behavioral assessment instruments will include:

- Liverpool Osteoarthritis in Dogs (LOAD) (243)
- Canine Frustration Questionnaire (CFQ)
- (135)
- Dog Impulsivity Assessment Scale (DIAS) (244)

#### 5.3.3 Protocol

Owners will complete questionnaires at baseline, mid-intervention (weeks 4–6), and final assessment (weeks 8–12). Scoring procedures will be performed using blinded assessment methods when feasible.

#### 5.3.4 Exploratory Outcomes

Potential exploratory findings may include reduced LOAD scores consistent with improved pain-associated function and mobility. In subsets of dogs with elevated baseline behavioral dysregulation, modest reductions in frustration-associated behaviors or changes in DIAS-related measures may also occur over time. Behavioral changes, when present, may demonstrate temporal relationships with gait, functional, or autonomic measures and could vary according to baseline severity, rehabilitation intensity, or treatment responsiveness.

#### 5.3.5 Falsification considerations

Findings that would argue against proposed CMRA-related interpretations include:

- behavioral changes occurring independently of any measurable functional or physiological adaptation,
- highly inconsistent behavioral responses across timepoints,
- worsening affective or frustration-associated behaviors despite consistent functional improvement across the cohort,
- or absence of any reproducible relationship between behavioral measures and other rehabilitation-associated outcomes.

Importantly, observed behavioral improvements alone would not constitute validation of the CMRA framework, as such changes may arise through multiple conventional mechanisms including pain reduction, improved mobility, altered owner perception, enhanced conditioning, or generalized improvement in quality of life.

### 5.4 Exploratory convergence across behavioral, autonomic, and functional domains

Within the CMRA framework, rehabilitation-associated improvements in autonomic measures, gait-related function, and pain-associated behavioral outcomes may demonstrate partially convergent patterns over time. In some individuals with elevated baseline behavioral dysregulation or stress-related responses, exploratory changes in frustration-associated behaviors or impulsivity-related measures may also occur alongside functional rehabilitation progress.

Patterns of partial convergence or divergence across domains may provide insight into the complexity and heterogeneity of chronic pain adaptation. For example, some dogs may demonstrate measurable gait improvement without substantial behavioral change, whereas others may exhibit behavioral or autonomic adaptation that exceeds detectable biomechanical recovery. Such variability may reflect differences in disease severity, pain chronicity, behavioral resilience, autonomic responsiveness, treatment exposure, or other conventional physiological and environmental factors.

Conversely, complete absence of reproducible relationships among behavioral, autonomic, and functional outcome measures across longitudinal assessment periods would weaken proposed CMRA-related interpretations and support the possibility that these domains operate largely independently within the context of rehabilitation-associated improvement.

Importantly, any observed convergence across domains would not constitute direct validation of the CMRA framework or proposed electromagnetic mechanisms, as multimodal rehabilitation outcomes may arise through multiple overlapping biological and behavioral processes.

## 6 Proposed mechanistic interpretation

### 6.1 Peripheral Tissue Adaptation and Bioenergetic Responses

#### 6.1.1 Phase 1 (minutes to hours)

Interventions such as photobiomodulation (PBM), pulsed electromagnetic field therapy (PEMF), electroacupuncture, therapeutic exercise, and myofascial interventions may produce early physiological effects involving mitochondrial metabolism, tissue perfusion, inflammatory signaling, autonomic responsiveness, connective tissue mobility, and mechanotransduction processes. Acute rehabilitation-associated changes may include reduced pain sensitivity, altered movement guarding, improved tissue mobility, and transient autonomic changes (80, 82, 241).

#### 6.1.2 Phase 2 (hours to days)

Over subsequent hours to days, rehabilitation interventions may contribute to modulation of inflammatory signaling, reduction in nociceptive burden, improvements in tissue mobility, and gradual restoration of movement variability and sensorimotor adaptation. Changes in mitochondrial metabolism, tissue hydration, circulation, and autonomic regulation may further contribute to functional improvement during this phase (80, 88, 241).

#### 6.1.3 Phase 3 (days to weeks)

Across longer rehabilitation intervals, progressive loading, therapeutic exercise, and regenerative interventions may support mitochondrial biogenesis, extracellular matrix remodeling, connective tissue adaptation, neuromuscular conditioning, and improved movement coordination through established physiological mechanisms including PGC-1*α*-associated pathways. These adaptations may contribute to improved gait stability, movement confidence, behavioral engagement, and functional recovery (88, 91, 92).

Within the CMRA framework, these phased physiological responses are interpreted as potentially interacting across biomechanical, autonomic, inflammatory, interoceptive, and affective-motor systems. Proposed electromagnetic contributions to these processes, however, remain theoretical and incompletely validated.

### 6.2 Multilevel physiological and sensorimotor adaptation

#### 6.2.1 Local tissue adaptation

Rehabilitation interventions may contribute to improvements in local tissue physiology through reductions in inflammatory burden, altered nociceptive input, improved circulation, enhanced tissue mobility, and changes in mitochondrial metabolism (80, 88). Within the CMRA framework, these physiological adaptations are hypothesized to potentially support more stable interoceptive and sensorimotor processing during chronic pain recovery.

#### 6.2.2 Myofascial and regional integration

Connected musculofascial regions may demonstrate coordinated changes in movement behavior, tissue loading, proprioceptive responsiveness, and neuromuscular adaptation during rehabilitation (207). These distributed changes may arise through overlapping biomechanical, autonomic, inflammatory, and sensorimotor mechanisms rather than isolated local tissue effects alone.

#### 6.2.3 Spinal processing and nociceptive modulation

Reductions in peripheral nociceptive burden and improved movement variability may influence spinal pathways involved in pain modulation and sensorimotor integration. Rehabilitation-associated changes in dorsal horn sensitization, wide dynamic range neuron activity, and afferent signaling may contribute to altered pain responsiveness and movement adaptation during recovery (245).

#### 6.2.4 Thalamic and supraspinal integration

Changes in peripheral input, autonomic state, movement behavior, and interoceptive processing may also influence thalamic and supraspinal systems involved in sensory integration and affective-motor regulation (246). Within the CMRA framework, these interactions are interpreted as part of broader physiological adaptation rather than evidence of validated large-scale electromagnetic coordination mechanisms.

### 6.3 Cingulate-Mediated affective and motor adaptation

#### 6.3.1 ACC-related affective adaptation

Reductions in nociceptive burden and movement-related distress during rehabilitation may influence anterior cingulate cortex (ACC)-related affective processing, threat appraisal, and autonomic regulation (213, 214, 216, 219). Improvements in pain-associated function and movement confidence could potentially correlate with reduced stress responsiveness and altered behavioral salience associated with painful movement. In some individuals with elevated baseline affective or stress-related dysregulation, broader behavioral adaptation may also occur over time.

#### 6.3.2 MCC-related motor adaptation

Improved movement variability, proprioceptive integration, and reduced pain-related guarding may support more efficient movement planning and motor adaptation involving midcingulate cortex (MCC)-related systems (41, 223–225). Changes in sensorimotor processing may contribute to increased movement confidence, altered gait strategies, and reductions in maladaptive protective behaviors during recovery.

#### 6.3.3 Autonomic regulation

Changes in pain burden, movement behavior, and stress responsiveness may also influence autonomic regulation through distributed cortical, subcortical, and peripheral mechanisms. Within the CMRA framework, physiological adaptation during rehabilitation may correlate with shifts toward more stable autonomic balance, potentially reflected in exploratory measures such as medial caruncle thermography (216).

#### 6.3.4 Adaptive updating of interoceptive and behavioral processing

Chronic pain states involve ongoing interactions among interoception, behavioral learning, autonomic regulation, nociceptive processing, and movement-related expectation (247, 248). Within the CMRA framework, rehabilitation-associated improvements in tissue function, movement behavior, and physiological regulation are hypothesized to potentially contribute to adaptive updating of affective-motor and interoceptive processing over time. Proposed relationships involving broader bioelectrical or electromagnetic organizational mechanisms remain speculative and experimentally unresolved.

### 6.4 Behavioral expression

Rehabilitation-associated physiological and behavioral adaptation may be reflected through observable changes in movement behavior, activity patterns, autonomic regulation, and quality of life. Potential findings may include improved gait symmetry, reduced movement variability, increased voluntary activity, enhanced movement confidence, and improved participation in previously avoided behaviors.

In some individuals with elevated baseline stress-related or frustration-associated behaviors, additional changes in behavioral flexibility, activity engagement, frustration tolerance, or impulse-related measures may also occur over time. Within the CMRA framework, such changes are interpreted as potentially reflecting interactions among pain reduction, autonomic adaptation, movement-related confidence, interoceptive processing, and affective-motor regulation during rehabilitation.

Importantly, these behavioral outcomes are non-specific and may arise through multiple overlapping mechanisms including pain reduction, improved conditioning, altered environmental interaction, enhanced mobility, or generalized improvement in well-being.

## 7 Exploratory predictions and potential falsification criteria

### 7.1 Exploratory prediction framework

#### 7.1.1 Test 1: multimodal convergence

Exploratory Prediction: Dogs receiving multimodal rehabilitation may demonstrate partially convergent changes across infrared thermography (IRT), gait-related measures, and pain-associated behavioral assessments over time.

Findings Potentially Inconsistent With the Framework: Absence of reproducible relationships across domains, highly divergent longitudinal trajectories, or complete independence among behavioral, autonomic, and biomechanical measures across repeated assessments.

#### 7.1.2 Test 2: differential responses across rehabilitation approaches

Exploratory Prediction: Rehabilitation approaches emphasizing multimodal tissue mobilization, movement variability, and sensorimotor engagement may demonstrate different temporal or functional response patterns compared with more limited intervention strategies.

Findings Potentially Inconsistent With the Framework: No observable differences in outcome trajectories across rehabilitation approaches, absence of reproducible associations between intervention intensity and measured outcomes, or highly inconsistent response patterns across individuals.

#### 7.1.3 Test 3: distributed functional effects

Exploratory Prediction: Some rehabilitation interventions may be associated with functional or behavioral changes extending beyond the immediately treated anatomical region, potentially reflecting distributed biomechanical, autonomic, sensorimotor, or behavioral adaptation.

Findings Potentially Inconsistent With the Framework: Strictly localized responses across assessments with no evidence of broader functional adaptation beyond directly treated tissues.

#### 7.1.4 Test 4: rehabilitation exposure and outcome relationships

##### 7.1.4.1 Exploratory prediction

Greater rehabilitation exposure or treatment intensity may correlate with larger or more sustained improvements across functional, autonomic, or behavioral outcome measures in some individuals.

##### 7.1.4.2 Findings potentially inconsistent with the framework

Absence of observable relationships between rehabilitation exposure and outcomes, highly inconsistent response patterns, or lack of measurable longitudinal adaptation despite repeated intervention.

#### 7.1.5 Test 5: differential temporal responses across modalities

##### 7.1.5.1 Exploratory prediction

Different rehabilitation modalities may demonstrate varying temporal response patterns across autonomic, behavioral, and functional outcome measures.

##### 7.1.5.2 Findings potentially inconsistent with the framework

Identical temporal response patterns across intervention types regardless of treatment characteristics or physiological targets.

Importantly, these exploratory predictions are not intended as definitive validation criteria for the CMRA framework. Observed relationships among behavioral, autonomic, and functional outcomes may arise through multiple overlapping physiological and rehabilitation-related mechanisms. Conversely, absence of predicted relationships would weaken aspects of the framework and may suggest that proposed systems-level interactions are less robust or mechanistically different than hypothesized.

### 7.2 Secondary predictions

- Baseline fascial dysfunction severity may correlate with rehabilitation response magnitude.
- Temporal sequencing may occur in which autonomic changes emerge first, followed by gait-related improvements and subsequently pain-linked behavioral adaptation. In individuals with baseline affective dysregulation, affective-motivational improvements may follow a parallel or delayed timeline.
- Functional improvements may occur despite persistent structural abnormalities.
- Rehabilitation effects may propagate more effectively along connected myofascial regions than across anatomically unrelated areas.
- Placenta-derived products may demonstrate rapid local physiological effects with broader functional changes in some individuals.
- Measures associated with interoceptive processing, autonomic regulation, and functional adaptation may demonstrate partial inter-correlation across assessments.
- Domain Selectivity of CMRA-Associated Effects

Emerging evidence in pain neuroscience and rehabilitation suggests that central adaptations are often domain-selective rather than globally expressed (6, 249, 250). Within the CMRA framework, nociceptive, proprioceptive, and autonomic processes may demonstrate greater sensitivity to chronic musculoskeletal dysfunction than affective-motivational systems in some individuals. Potential contributors to such selectivity may include the anatomical distribution of fascial and musculoskeletal dysfunction, differences in cingulate subregional connectivity, and individual variation in affective regulation, stress resilience, and behavioral coping strategies. Recognition of domain selectivity improves the explanatory precision and falsifiability of the CMRA framework by distinguishing broader physiological predictions from more conditional affective-motivational predictions.

### 7.3 Integration with neuroscience frameworks

The CMRA framework is conceptually informed by several established and emerging perspectives relevant to chronic pain, interoception, movement behavior, and physiological adaptation, including predictive processing and predictive coding models (251, 252), allostatic load frameworks (253, 254), interoceptive inference theories (255, 256), embodied cognition approaches (257, 258), and biotensegrity concepts (173, 259).

Rather than proposing a definitive unification of these frameworks, the CMRA model is intended as an exploratory systems-level hypothesis integrating aspects of biomechanics, autonomic regulation, interoceptive processing, affective-motor adaptation, and rehabilitation physiology within chronic musculoskeletal disease and recovery.

Importantly, many proposed relationships within the CMRA framework remain theoretical and experimentally unresolved, particularly those involving large-scale electromagnetic organizational mechanisms.

## 8 Discussion

### 8.1 Veterinary rehabilitation implications

Building upon the experimental literature reviewed throughout this manuscript, if aspects of the CMRA framework are supported through future experimental investigation, the model could encourage broader systems-level approaches to rehabilitation assessment and treatment interpretation in veterinary medicine. Rather than focusing exclusively on peripheral structural pathology, rehabilitation strategies may increasingly consider interactions among movement behavior, autonomic regulation, interoceptive processing, affective-motivational adaptation, and functional recovery.

Within this framework, multimodal rehabilitation approaches integrating manual therapy, therapeutic exercise, neuromodulatory interventions, regenerative medicine strategies, and behavioral assessment may provide opportunities to explore how diverse physiological systems interact during chronic pain recovery. The CMRA model also supports consideration of behavioral and autonomic measures alongside conventional biomechanical outcomes when evaluating rehabilitation-associated change.

Interventions such as photobiomodulation, electroacupuncture, pulsed electromagnetic field therapy, regenerative medicine approaches, and myofascial rehabilitation techniques may influence overlapping physiological processes involved in mitochondrial metabolism, inflammatory regulation, autonomic function, tissue mobility, movement confidence, and sensorimotor adaptation (15, 61, 260–263). Within the CMRA framework, proposed electromagnetic organizational mechanisms linking these responses remain theoretical and incompletely validated.

The proposed multimodal assessment approach—integrating autonomic measures (infrared thermography), biomechanical analysis (quantitative gait assessment), and behavioral evaluation (validated questionnaires)—may support more comprehensive characterization of rehabilitation-associated adaptation across multiple functional domains.

Future research may help determine whether individual variability in tissue state, behavioral responsiveness, autonomic regulation, pain chronicity, or movement adaptation contributes to differing rehabilitation responses across patients. Such work could ultimately support more individualized rehabilitation planning without requiring deterministic assumptions regarding large-scale electromagnetic signaling mechanisms.

#### 8.2 Relevance for human chronic pain and neurorehabilitation

The CMRA framework may have translational relevance for future investigation in chronic pain, neurorehabilitation, sports medicine, and movement-related disorders. In chronic pain populations—including individuals with myofascial pain syndromes, fibromyalgia, and chronic widespread pain—multimodal assessment strategies integrating autonomic, behavioral, and biomechanical measures may help explore relationships among pain adaptation, movement behavior, interoceptive processing, and rehabilitation-associated change (1, 83, 237).

Within this exploratory framework, interventions involving myofascial rehabilitation, therapeutic exercise, neuromodulatory modalities, and regenerative medicine approaches could potentially be evaluated for their effects on functional adaptation, autonomic regulation, pain-associated behavior, and movement-related confidence over time. Comparative findings across species may help clarify whether broader chronic pain physiological patterns relevant to rehabilitation are conserved across humans and companion animals, thereby informing bidirectional translational investigation (264–266).

The CMRA framework also offers a conceptual perspective for interpreting aspects of myofascial pain and movement dysfunction through interactions among connective tissue mechanics, autonomic regulation, sensorimotor adaptation, interoceptive processing, and chronic pain-related behavioral change. Proposed electromagnetic organizational mechanisms underlying these interpretations, however, remain theoretical and experimentally unresolved.

In sports medicine and performance rehabilitation contexts, multimodal assessment approaches incorporating movement analysis, behavioral adaptation, autonomic measures, and tissue-function evaluation may complement conventional biomechanical and structural criteria during recovery and return-to-activity decision-making. Future research may help determine whether broader physiological or behavioral patterns contribute to rehabilitation variability across individuals.

Similarly, multimodal rehabilitation approaches—including regenerative medicine strategies when appropriate—may influence rehabilitation outcomes through interacting effects on tissue healing, inflammation, extracellular matrix remodeling, mitochondrial metabolism, and functional recovery (110, 116, 118, 119). Within the CMRA framework, such effects are interpreted within a broader rehabilitation context rather than as evidence for validated large-scale electromagnetic signaling mechanisms.

### 8.3 Limitations and important caveats

Several important methodological and conceptual limitations warrant acknowledgment. The proposed assessment battery does not directly measure cingulate activity, electromagnetic organization, or interoceptive processing. Rather, the framework interprets behavioral, autonomic, and biomechanical measures as indirect physiological correlates that may relate to broader rehabilitation-associated adaptation. While this approach improves clinical feasibility and translational applicability, it cannot establish causality or directly validate proposed CMRA-related mechanisms.

The resonome and related concepts involving large-scale physiological organization remain theoretical and experimentally unresolved constructs rather than directly measurable biological entities in current clinical practice. Although individual physiological components relevant to the framework—including mitochondrial metabolism, connective tissue properties, autonomic regulation, and neural oscillatory activity—can be studied independently, the broader systems-level relationships proposed by the CMRA framework remain speculative and require substantial empirical validation.

Current clinical assessment of fascial tissue quality also remains limited. Palpatory evaluation is inherently subjective and examiner-dependent despite clinical training and experience. Imaging approaches such as musculoskeletal ultrasound may provide additional assessment of fascial thickness, echogenicity, tissue mobility, and elasticity, although standardized protocols and normative reference data for canine fascial structures remain incompletely developed (82, 206)

The naturalistic rehabilitation setting introduces substantial heterogeneity and potential confounding variables including differences in age, breed, disease chronicity, medication use, comorbidities, home environment, owner compliance, and rehabilitation exposure. While this variability may improve ecological validity and translational relevance, it complicates mechanistic interpretation and limits causal inference.

Behavioral outcome measures also depend partly on owner observation and interpretation, introducing potential reporting bias despite the use of validated instruments (134–136). Future studies incorporating objective behavioral coding systems, automated activity monitoring, wearable physiologic sensors, or additional autonomic biomarkers may strengthen assessment reliability.

Similarly, the proposed assessment schedule provides limited temporal resolution for evaluating rehabilitation-associated adaptation over time. More frequent longitudinal assessment of gait, behavioral, and autonomic measures may better characterize response trajectories during rehabilitation.

Finally, the CMRA framework proposes interactions across multiple biological scales involving tissue mechanics, autonomic regulation, mitochondrial metabolism, sensorimotor processing, and affective-motor adaptation. While such complexity may reflect aspects of chronic pain physiology and rehabilitation biology, it also creates challenges for isolating specific causal mechanisms. Carefully controlled experimental studies targeting individual physiological domains will therefore be necessary to determine which aspects of the framework, if any, are empirically supported or refuted.

### 8.4 Future directions

Emerging Physiological Measurement Approaches Future research may benefit from increasingly sophisticated multimodal approaches for investigating relationships among connective tissue physiology, mitochondrial metabolism, autonomic regulation, neural activity, and rehabilitation-associated adaptation. Potentially relevant technologies may include advanced imaging, autonomic monitoring, wearable physiologic sensors, ultrasound elastography, bioimpedance methods, and emerging approaches capable of characterizing subtle physiological activity across tissues (129, 131, 181).

Although such methods may help evaluate aspects of the CMRA framework, no current technology directly measures “resonome organization” or proposed large-scale electromagnetic coordination mechanisms *in vivo*. Future work will therefore require careful operationalization, reproducibility testing, and distinction between speculative theoretical constructs and empirically measurable physiological variables.

Neuropsychiatric and Chronic Pain Applications Future interdisciplinary research may also explore whether relationships among chronic pain, interoception, autonomic regulation, movement behavior, affective processing, and somatic rehabilitation are relevant across neuropsychiatric and chronic pain conditions. Disorders involving chronic stress, affective dysregulation, altered body awareness, or persistent pain—including depression, anxiety disorders, and PTSD—may provide useful contexts for studying interactions among physiological regulation, behavioral adaptation, and embodied aspects of chronic illness (2, 247, 248).

Within this exploratory framework, multimodal rehabilitation strategies may be evaluated for broader effects on function, stress responsiveness, behavioral adaptation, autonomic regulation, and quality of life. Proposed electromagnetic organizational mechanisms underlying these interpretations remain theoretical and experimentally unresolved.

Future neuroimaging, autonomic, and behavioral studies may help clarify whether rehabilitation-associated physiological changes correlate with altered affective-motor processing or interoceptive adaptation across chronic pain and neuropsychiatric populations. Such findings could support broader investigation into systems-level rehabilitation biology without constituting direct validation of the CMRA framework itself.

## 9 Conclusion

This manuscript introduces the Cingulate–Mitochondrial–Resonome Axis (CMRA) as an exploratory systems-level hypothesis framework integrating concepts from rehabilitation science, interoception, autonomic regulation, chronic pain neuroscience, movement behavior, and affective-motor processing within chronic musculoskeletal disease. Drawing conceptually from the FoK–FIP framework and related theories involving embodied physiology and interoceptive processing, the CMRA proposes that rehabilitation-associated adaptation may involve interactions among tissue mechanics, mitochondrial metabolism, autonomic regulation, sensorimotor integration, and cingulate-mediated behavioral processes.

Rather than focusing exclusively on peripheral structural pathology, the CMRA framework encourages consideration of how multimodal rehabilitation interventions may influence broader physiological and behavioral processes relevant to chronic pain adaptation and functional recovery. The framework also proposes a multimodal assessment approach incorporating autonomic measures, biomechanical analysis, and behavioral evaluation as exploratory methods for studying rehabilitation-associated change across multiple functional domains.

Importantly, many proposed mechanisms within the CMRA framework—particularly those involving large-scale electromagnetic organizational processes—remain theoretical and experimentally unresolved. Accordingly, the CMRA is presented not as an established mechanistic model, but as a hypothesis-generating framework intended to stimulate interdisciplinary investigation and future experimental testing.

If aspects of the framework are supported through future research, the CMRA may contribute to broader understanding of how rehabilitation influences interactions among movement behavior, autonomic regulation, interoceptive processing, affective adaptation, and chronic pain recovery across species. More broadly, the CMRA framework highlights the potential value of systems-level, multimodal approaches for investigating rehabilitation-associated adaptation and provides a hypothesis-generating foundation for future interdisciplinary research.

## Data Availability

The original contributions presented in the study are included in the article/Supplementary Material, further inquiries can be directed to the corresponding author.
